# Improvements to Healthspan Through Environmental Enrichment and Lifestyle Interventions: Where Are We Now?

**DOI:** 10.3389/fnins.2020.00605

**Published:** 2020-06-12

**Authors:** Nicholas J. Queen, Quais N. Hassan, Lei Cao

**Affiliations:** ^1^Department of Cancer Biology and Genetics, College of Medicine, The Ohio State University, Columbus, OH, United States; ^2^The Ohio State University Comprehensive Cancer Center, Columbus, OH, United States; ^3^Medical Scientist Training Program, College of Medicine, The Ohio State University, Columbus, OH, United States

**Keywords:** environmental enrichment, healthy aging, healthspan, BDNF, hypothalamus, HPA axis, lifespan

## Abstract

Environmental enrichment (EE) is an experimental paradigm that is used to explore how a complex, stimulating environment can impact overall health. In laboratory animal experiments, EE housing conditions typically include larger-than-standard cages, abundant bedding, running wheels, mazes, toys, and shelters which are rearranged regularly to further increase stimulation. EE has been shown to improve multiple aspects of health, including but not limited to metabolism, learning and cognition, anxiety and depression, and immunocompetence. Recent advances in lifespan have led some researchers to consider aging as a risk factor for disease. As such, there is a pressing need to understand the processes by which healthspan can be increased. The natural and predictable changes during aging can be reversed or decreased through EE and its underlying mechanisms. Here, we review the use of EE in laboratory animals to understand mechanisms involved in aging, and comment on relative areas of strength and weakness in the current literature. We additionally address current efforts toward applying EE-like lifestyle interventions to human health to extend healthspan. Although increasing lifespan is a clear goal of medical research, improving the quality of this added time also deserves significant attention. Despite hurdles in translating experimental results toward clinical application, we argue there is great potential in using features of EE toward improving human healthy life expectancy or healthspan, especially in the context of increased global longevity.

## Introduction

### Environmental Enrichment

Environmental enrichment (EE) is a laboratory animal housing model that combines complex physical, social, cognitive, motor, and somatosensory stimuli to elicit improvements in systemic metabolism, immunity, and behavior (Nithianantharajah and Hannan, 2006). Although EE housing protocols vary between labs (Nithianantharajah and Hannan, 2006; Sztainberg and Chen, 2010; Slater and Cao, 2015), they achieve similar and reproducible results. Common experimental methods incorporate toys, shelters, running wheels, increased levels of bedding and housing surface area, and varied social networks, (Nithianantharajah and Hannan, 2006; Sztainberg and Chen, 2010; Slater and Cao, 2015) and the physical components of EE are frequently rearranged for an added level of stimulation. In contrast, standard housing environments (SE) incorporate limited bedding, fewer animals, and a nestlet inside a much smaller and unchanging shoebox-sized cage.

EE elicits numerous biological changes that appear quickly and are maintained throughout the housing period. One particularly impactful phenotypic feature of EE is the activation of a brain-adipose axis, dubbed the hypothalamic-sympthoneural-adipocyte (HSA) axis (Cao et al., 2010, 2011; McMurphy et al., 2018b). An upstream mediator, brain-derived neurotrophic factor (BDNF), is upregulated in as soon as 2 weeks within the EE paradigm (Cao et al., 2010). The induction of BDNF – whether through EE or other experimental means – initiates numerous downstream biological changes in peripheral tissues directly or indirectly through regulating sympathetic tone to the adipose tissues, manifesting in: (1) reduced weight gain – despite increased food intake (Cao et al., 2009, 2010, 2011; Liu et al., 2014b; McMurphy et al., 2018a, b; Ali et al., 2019, 2020; Foglesong G. et al., 2019; Queen et al., 2020), (2) improved glycemic control (Cao et al., 2009, 2010, 2011; Liu et al., 2014b; During et al., 2015; McMurphy et al., 2018a, b; Ali et al., 2019, 2020; Foglesong G.D. et al., 2019; Queen et al., 2020), (3) reduced circulating leptin (Cao et al., 2009, 2010, 2011; Nachat-Kappes et al., 2012; Liu et al., 2014b; McMurphy et al., 2018a, b; Foglesong G. et al., 2019; Ali et al., 2020; Queen et al., 2020), (4) increased circulating adiponectin (Cao et al., 2009, 2010; Nachat-Kappes et al., 2012; Liu et al., 2014a; McMurphy et al., 2018a, b; Foglesong G.D. et al., 2019; Queen et al., 2020), (5) decreased adiposity and increased total lean mass (Cao et al., 2009, 2010, 2011; During et al., 2015; McMurphy et al., 2018a, b; Ali et al., 2019, 2020; Queen et al., 2020), (6) adipose browning/beiging and elevated energy expenditure (Cao et al., 2010, 2011; During et al., 2015; McMurphy et al., 2018a, b), (7) reduced hepatosteatosis (Cao et al., 2010; Liu et al., 2014b; McMurphy et al., 2018a, b), and (8) suppression of cancer growth (Cao et al., 2010; Nachat-Kappes et al., 2012; Liu et al., 2014b; Li et al., 2015; Xiao et al., 2016; Foglesong G. et al., 2019; Meng et al., 2019). Furthermore, hypothalamic BDNF plays an important role in mediating the immunomodulating effects of EE through the sympathetic nervous system (SNS) and/or hypothalamic-pituitary-adrenal (HPA) axis (Xiao et al., 2016), as well as certain behavior changes such as increased locomotion and decreased anxiety/depression-like behavior (Cao et al., 2010; McMurphy et al., 2018a, b; Queen et al., 2020). Others have shown that peripheral administration of BDNF affects glucose utilization (Yamanaka et al., 2007b, 2008a), energy expenditure (Yamanaka et al., 2007a, 2008b), and food intake (Yamanaka et al., 2008a). Loss-of-function studies provide further supportive evidence for the systemic functions of BDNF; heterozygosity for *Bdnf* results in hyperphagia, obesity, and abnormal locomotor activity (Kernie et al., 2000; Rios et al., 2001). In humans, single nucleotide polymorphism (SNP) variants in the *BDNF* locus have been associated with higher body mass index (BMI) (Speliotes et al., 2010; Mou et al., 2015). Additional work has shown a negative correlation between body weight and plasma BDNF levels (Lommatzsch et al., 2005).

The ability of EE to improve overall behavioral and cognitive health has been widely recognized through BDNF-dependent and –independent mechanisms. Several experimental tools exist to assess anxiety, depression, and sociability. Behavioral and pharmacological manipulations have revealed that EE reduces anxiety-like (Benaroya-Milshtein et al., 2004; Sztainberg et al., 2010), and depression-like behaviors (Jha et al., 2011), and can increase sociability in models with abnormal social behavior (Queen et al., 2020). Further work has shown the ability of EE to improve cognition (Arendash et al., 2004; Jankowsky et al., 2005), and memory (Bruel-Jungerman et al., 2005; Prado Lima et al., 2018) in models of normal and abnormal neural function.

Combined, these brain-driven systemic changes yield an overall improvement of physical and mental health. In summary, EE is a valuable experimental model that allows researchers to understand the role of environmental influences on physiological and pathological processes. With EE, researchers ultimately elicit broad, systemic physiologic changes with easily identifiable phenotypes, demonstrating a clear brain-body connection in the context of both animal and human disease models. Accordingly, the EE paradigm is exceptionally well suited to study and modulate systemic processes like aging; the overlap of the two will be the focus of this review.

### Lifespan, Healthspan, and Aging

To understand the aging process, one first must understand the nuances between life span, life expectancy, and healthspan. Lifespan describes the time between an organism’s birth and its death, while life expectancy is an estimate of expected duration of life for an individual based on a statistical evaluation of the population. There has been recent debate (Dong et al., 2016; Olshansky, 2016; de Beer et al., 2017; Lenart and Vaupel, 2017; Rozing et al., 2017) about a biological maximum limitation on lifespan. The prevailing consensus suggests that any possible improvements to lifespan, and thus life expectancy, are likely to be difficult despite previous successes. This is because the most significant extensions of life expectancy achieved thus far have occurred by reductions of childhood mortality; further increases in life expectancy will be more difficult despite advances in medicine and technology because they must occur by extending the lives of older people (Olshansky, 2018).

Thus, a more feasible, and significantly more important problem to focus on would be extending healthspan. Broadly speaking, if lifespan is defined as the total length of time an organism spends alive, healthspan is the subset duration of time of an organism’s life spent in good health. One of the first academic references to healthspan was in a review on human aging by Rowe and Kahn in 1987 where they stated, “[A substantial] increase in health span, the maintenance of full function as nearly as possible to the end of life, should be the next gerontological goal” (Rowe and Kahn, 1987). In other words, an ideally maximized healthspan would be an organism living in perfect health until the exact moment of its death.

From an individual organism’s perspective, the imperfect reconciliation between lifespan and healthspan overall results in decreased functionality, diminished satisfaction, and increased pain and suffering. Furthermore, on a population scale, this discrepancy results in decreased societal productivity, and increased costs of healthcare. Thus, extensions of lifespan without extension of healthspan are likely to bring predominantly negative effects. Current work to study or expand healthspan has typically focused on increasing the “span,” that is the time someone spends healthy before declining. However, health is not a binary state of being, but instead a relative spectrum. As organisms progress through life, they not only have longer to receive injury, but they also experience a decline in general health and physiological function due to the normal aging process. Thus, efforts to evaluate or improve healthspan should also consider elements of improving general health; meaningful improvements to healthspan can and do occur without expansion of its duration.

Aging as a concept, despite an almost ubiquitous experience, has been notoriously difficult to define. Early definitions included “the progressive increase of risk of death over time” (Comfort, 1979), primarily linking aging to lifespan, and “a persistent decline in the age-specific fitness components of an organism due to internal physiological degeneration” (Rose, 1994), which indirectly considers healthspan. Rowe and Kahn defined successful aging as “low probability of disease and disease-related disability, high cognitive and physical functional capacity, and active engagement with life” (Rowe and Kahn, 1997). More recently, aging has been reviewed and re-defined (Holliday, 2006; Hayflick, 2007; Flatt, 2012; Rose et al., 2012; Kennedy et al., 2014; Gladyshev, 2016) with further and significant considerations toward positive aspects of aging, the heterogeneity of aging, and the feasibility of understanding and reversing the aging process. Additionally, much work has been done to establish and review the biochemical and cellular mechanisms of aging (Rattan, 2009; Salminen and Kaarniranta, 2009; Kenyon, 2010; van Deursen, 2014; Childs et al., 2015; Longo et al., 2015; Kauppila et al., 2017; Herranz and Gil, 2018) as well as potential pharmacological therapeutic options (Kaeberlein et al., 2015; Shetty et al., 2018; Fuellen et al., 2019; Vaiserman et al., 2019; Hodgson et al., 2020). We encourage interested readers to explore these reviews and others for more contextual understanding of aging.

There is still no consensus as to if aging is a deviation from normal biology or if it simply represents a range, or maturation, of normal biology. With many negative changes associated with aging, some call for aging to be classified as a disease (Bulterijs et al., 2015; De Winter, 2015). Others firmly believe that aging is not a disease (Hayflick, 2007; Rattan, 2009; Kennedy et al., 2014; Gavrilov and Gavrilova, 2017) or acknowledge it as is “neither disease nor non-disease” (Gladyshev and Gladyshev, 2016) or a “gray area between health and sickness” (Gems, 2015). Aging is a broad phenomenon, and variation of an aged phenotype within an individual organism, all organisms within a species, or even among all lifeforms, makes aging impossible to be reduced into a single definition or defined as a disease. Therefore, we take the perspective that aging is associated, but distinct from age-related diseases. In other words, while aging has a correlative, or potentially causative relationship with disease, and in many cases may be intertwined with specific disease states, it is not disease itself. Similarly, although healthspan differs from aging and age-associated disease, they are intricately linked, and improvements to age-related conditions result in improved healthspan.

**FIGURE 1:**
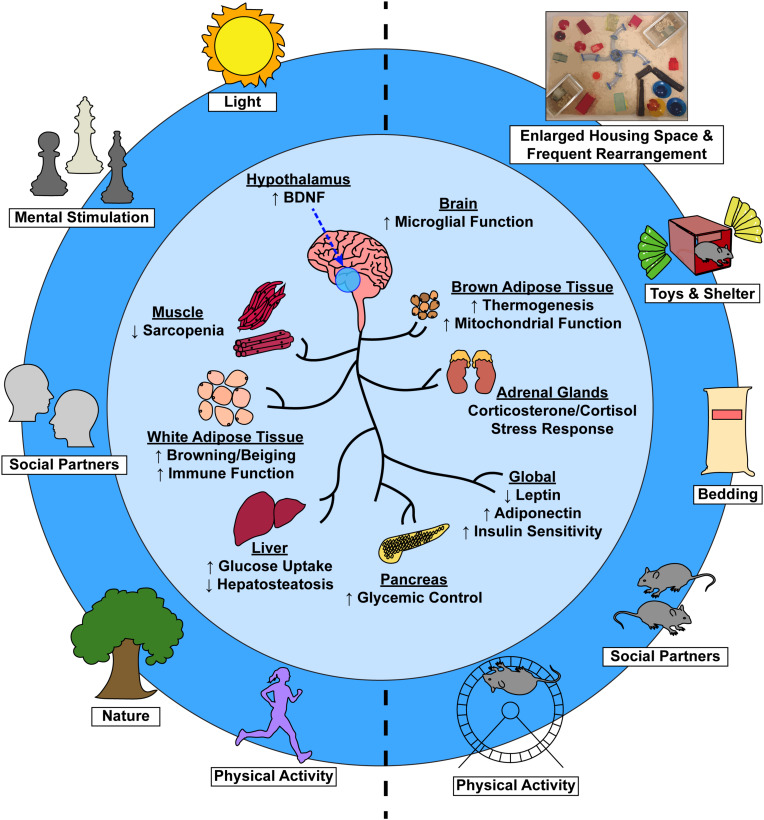
Environmental factors induce systemic physiological change via a brain-body connection. Complex EE stimuli in laboratory animals **(right)** and analogous stimuli in humans **(left)** contribute to not only improved psychological health, but to numerous tissue-specific and global physiological benefits as well **(center)**.

Currently, a significant portion of the aging research community wishes to elucidate how maximization of healthspan might occur. The EE model is uniquely positioned in healthspan research due to its ability to ameliorate or prevent many aspects of aging-related physiological decline. As such, this review will serve to discuss the work of our lab and others to highlight how EE can be a powerful tool to elucidate novel systemic and local mechanisms underlying age-related bodily change, disease, and health (Figure 1). While this is not the first review to summarize the benefits of enrichment across the lifespan – and is certainly not the first to mention gene-environment interactions on neurodegenerative disease (Nithianantharajah and Hannan, 2006; Laviola et al., 2008; Pang and Hannan, 2013; Mo et al., 2015; Shepherd et al., 2018), cognitive disorders (Ohline and Abraham, 2019; Rogers et al., 2019), and psychiatric disorders (Nithianantharajah and Hannan, 2006; Burrows et al., 2011; Rogers et al., 2019) – this review newly broaches the healthspan issue, highlights EE-healthspan work, and investigates the “translatability problem” of such research. Accordingly, we discuss the search for human lifestyle interventions that elicit EE-like mechanisms and further emphasize the importance of understanding conserved mechanisms and environmental factors that may contribute to healthy aging. With these insights, we hope both clinicians and researchers can better develop and implement therapeutic applications in the pursuit of increasing human healthspan.

## Biological Consequences of Aging and the Need for Improvements in Healthspan

To understand the interplay between environmental factors, aging, and healthspan, one must first consider the biological mechanisms implicated in the normal aging process. Here, we will present several age-modulated biological processes and consider how the brain-body connection contributes to the physical manifestation of aging.

### Cognitive and Behavioral Aspects of Aging

Age-related structural and functional alterations within the brain – including subtle synapse loss (Smith et al., 2000; Adams et al., 2010) in the absence of global reductions (Calhoun et al., 1998; Poe et al., 2001; Shi et al., 2007; Newton et al., 2008), reduced synaptic plasticity (Hatanpää et al., 1999), reduced spine density (Dickstein et al., 2013), dendritic regression (Dickstein et al., 2013), and increased neuroinflammation (Chen et al., 2016; Guillemot-Legris and Muccioli, 2017), among others – can result in decreased cognitive ability. With age, mental processing speed slows (Verhaeghen and Salthouse, 1997; Salthouse, 2000), memory fades (Spencer and Raz, 1995; Zacks et al., 2000), and executive function declines (Buckner, 2004), while some functions – including control over language – remain relatively stable. Though cognitive decline is common in aging, the rate and extent of decline is highly heterogeneous. For some, cognitive decline is so mild that it may be imperceptible, while for others, resultant diseases like dementia and Alzheimer’s are debilitating for patients and their families.

The aforementioned alterations in the central nervous system (CNS) can also result in altered behavior and emotionality. The elderly are at risk for many mental illnesses, including depression, anxiety, and schizophrenia. Decreased BDNF levels and mutations in genes associated with BDNF signaling have been associated with hippocampal dysfunction (Egan et al., 2003), memory impairment (Egan et al., 2003), increased risk for depression (Martinowich et al., 2007; Erickson et al., 2012), schizophrenia (Angelucci et al., 2005), and anxiety (Egan et al., 2003; Martinowich et al., 2007; Erickson et al., 2012). In contrast, physical activity has been linked to an increase in BDNF levels and a reversal of these pathologies (Lampinen et al., 2000; Laske et al., 2011).

### The Hypothalamic-Pituitary-Adrenal (HPA) Axis

During the aging process, the body is subject to various stressors that activate neuroendocrine signaling via the HPA axis. A bevy of evidence indicates that the HPA axis can contribute to biological aging, whether through dysregulated glucocorticoid secretion or alterations in the production of regulatory peptides across one’s lifespan (Sapolsky et al., 1986; Aguilera, 2011; Nehlin, 2017). Corticotropin-releasing hormone (CRH) is one actor within the HPA axis and is highly expressed within hypothalamic nuclei. CRH is an upstream regulator of pituitary adrenocorticotropic hormone (ACTH) synthesis and adrenal production of corticosterone/cortisol (Vale et al., 1981; Nehlin, 2017). While the literature is mixed on how aging alters CRH production, the predominant belief is that production increases with age (Sapolsky et al., 1986; Tizabi et al., 1992; Hauger et al., 1994; Meijer et al., 2005; Aguilera, 2011; Vitale et al., 2013; Nehlin, 2017), perhaps due to inhibition of glucocorticoid- and mineralcorticoid-driven negative feedback loops (Vitale et al., 2013). The regulation of HPA axis activity is further complicated by crosstalk that occurs between the central HPA axis and peripheral tissues, including adipose, muscle, and liver tissue (McEwen, 2007; Iwen et al., 2008; Petrescu et al., 2018). Accordingly, dysregulation of the HPA axis is associated with various maladies, including Alzheimer’s disease (Swaab et al., 2005; Green et al., 2006), metabolic syndrome (Chrousos, 2000; Moraitis et al., 2017), depression (De Kloet et al., 2005; Swaab et al., 2005), mood disorders (De Kloet et al., 2005; Swaab et al., 2005), and other forms of mental illness. In sum, HPA signaling is multifaceted due to its role in central and peripheral mechanisms (Spencer et al., 2015); alterations of this important neuroendocrine axis can lead to many age-associated diseases, the mechanisms of which are explored in greater detail in the following valuable reviews (Chahal and Drake, 2007; Aguilera, 2011; Murri et al., 2014).

### Metabolic Function

A decrease in metabolic function accompanies the natural aging process, due in part to hypothalamic neuroinflammation (Guillemot-Legris and Muccioli, 2017; Cai and Khor, 2019). Murine models experience an increase in body mass through middle and old age (Turturro et al., 1999); such body composition change is to increases in total fat mass and occurs despite concurrent decreases in lean mass (Houtkooper et al., 2011; Zhu et al., 2019). This change in adiposity is thought to result from decreased physical activity (White et al., 2016) and age-related alterations in lipolysis (Mennes et al., 2013), fatty acid oxidation (Houtkooper et al., 2011), and mitochondrial function (Katic et al., 2007). Aged models display impairments in glycemic control, likely due to an underlying reduction in pancreatic beta cell function or the development of peripheral insulin resistance (Houtkooper et al., 2011; McMurphy et al., 2018a, b; Abarkan et al., 2019). Leptin and adiponectin are two adipokines that have been linked to metabolic health, with the former being positively correlated and the latter being negatively correlated to adipose mass (Lutz and Quinn, 2012). Accordingly, leptin and adiponectin play roles in energy expenditure, thermogenesis, lipid metabolism, and insulin resistance (Scarpace et al., 2000; Gil-Campos et al., 2004) and have been shown to be modulated by the aging process. With age, an increase in leptin and a decrease in adiponectin occurs (Palmer and Kirkland, 2016). As this might suggest, increased levels of adiponectin have been positively correlated to longevity and good metabolic health (Atzmon et al., 2008; Palmer and Kirkland, 2016) whereas the development of central and peripheral leptin resistance in aged models contributes to obesity and other metabolic dysfunction resulting in numerous further comorbidities (Scarpace et al., 2000, 2001).

### Immune Function

The aging process has been shown by many to influence immune function. Elsewhere, others have summarized the aging-immune interplay to a great extent (Weiskopf et al., 2009; Pereira and Akbar, 2016; Nikolich-Žugich, 2018; Cai and Khor, 2019), however, this review would be remiss to avoid touching this subject completely. Age-related deterioration of the CNS coincides with reduced peripheral organ dysfunction and is thought to result in many of the systemic age-related changes in metabolism, immunity, and behavior. Microglial cells are CNS specific resident macrophage-like immune cells, acting as surveyors to sense immunogenic stimuli or tissue injury. Upon insult, microglia produce proinflammatory mediators, leading to further immune signaling, phagocytosis of cellular debris, and presentation of antigens to the immune system (Guillemot-Legris and Muccioli, 2017). Additionally, through BDNF signaling, microglia contribute to synaptic plasticity (Ferrini and De Koninck, 2013; Parkhurst et al., 2013). Aging-associated microglial dysfunction is characterized by hyper-reactivity to inflammatory stimuli, higher basal levels of cytokine expression, reduced surveilling activity, shortened process length, and slower movement (Hefendehl et al., 2014; Norden et al., 2015; Ziebell et al., 2015). Functional decline of microglia is associated with neurodegenerative disease and peripheral tissue dysfunction (Chen et al., 2016; Spittau, 2017).

### Aging and Epigenetic Change

Environmental stimuli are thought to alter the epigenome, thus influencing healthspan and lifespan (Benayoun et al., 2015; Sen et al., 2016). Recent work has suggested that biological aging proceeds under the control of a so-called “epigenetic clock”; in this model, DNA methylation levels are used as a correlative biomarker of aging (Horvath, 2013; Horvath and Raj, 2018). This epigenetic clock can be influenced by environmental exposures and lifestyle factors like diet, physical activity, education, smoking, and alcohol consumption (Hannum et al., 2013; Quach et al., 2017; Horvath and Raj, 2018). Molecular and functional alterations are associated with one’s epigenetic regulation state and accordingly, age-related physiological decline has been associated with epigenetic regulation. Alterations in promoter methylation have been observed in aged samples for genes involved in hippocampal function (Haberman et al., 2012) and memory storage (Penner et al., 2011). Some evidence suggests that Alzheimer’s disease is modulated by environmental factors – including diet and physical activity – and associated epigenetic regulation (Chouliaras et al., 2010; Griñán-Ferré et al., 2018). Other age-related processes and disease states have been tied to epigenetic clock shifts, including metabolic syndrome (Horvath et al., 2014), Parkinson’s disease (Horvath and Ritz, 2015), cardiovascular disease (Perna et al., 2016), and cancer (Perna et al., 2016). Furthermore, a recent commentary (Hannan, 2020) highlights the potential for therapeutics mimicking some of the effects of EE and lifestyle interventions by targeting epigenetic factors. Altogether, these studies highlight the importance of gene-environment interactions, further highlighting the role that exogenous factors can play in healthspan and the aging process.

## Environmental Enrichment and Aging

Human longitudinal aging research is challenging due to our species’ lifespan length. Mice are the mammalian model of choice due to their short lifespan, homology, size, and ease of rearing. Laboratory mice have mean lifespans of 2–3 years in well-controlled housing conditions (Turturro et al., 1999). The murine aging process mirrors that of humans and allows researchers to perform work on the biology of aging in a comparatively short time span. It is important to note that some murine age-related observations have been found to be strain- and source-dependent; special considerations must be made when comparing and designing such experiments (Turturro et al., 1999; Brayton et al., 2012).

As previously detailed, aging results in a myriad of physical changes in both humans and mammalian models that, when combined, can manifest in disease. EE-induced improvements in systemic metabolism, immunity, and behavior run counter to many age-associated deficits. Accordingly, researchers have investigated whether the EE model can ameliorate or perhaps prevent age-related disease. Here, we will discuss the work of our lab and others to highlight how EE can be a powerful tool to elucidate novel systemic and local mechanisms underlying age-related bodily change, disease, and health.

### Promoting Healthy Aging Through EE

Spurred by observations of EE-driven improvements in the systemic metabolic, immunity, and behavior in young mice, experiments by our lab investigated the ability of EE to promote healthy aging in aged mice (McMurphy et al., 2018b). When initiated at middle-age (10 months old), short-term EE (6 weeks) was able to activate the hypothalamic-sympathoneural-adipocyte (HSA) axis in a similar fashion to young counterparts. Consistent with previous work, we observed an upregulation of hypothalamic *Bdnf* as well as associated improvements in metabolism and related molecular pathways. These results acted as a proof-of-concept for the use of EE in aged models, indicating that even a short exposure of EE – after lifelong housing in standard conditions – could yield a meaningful phenotypic shift.

Having shown that EE provides short-term benefits in both young and old mice, we were especially interested in identifying the potential for lasting effects, as the utility of these benefits would be questionable if they were temporary. We assessed the ability of EE to impart lasting and consistent phenotypic changes over the murine lifespan, thus increasing healthspan (McMurphy et al., 2018b) by enrolling mice with no history of enrichment at 10 months of age in either standard housing or EE for an additional 12 months. As in previous experiments, mice in EE experienced an upregulation of hypothalamic *Bdnf*, as well as improvements in global metabolism and behavior, Mice exhibited decreased fat mass with increased lean mass, improved glycemic control, reduced hepatosteatosis, increased locomotion, and decreased anxiety over a year-long period, suggesting that EE provides durable and significant improvements to healthspan in laboratory mice and promote healthy aging. In a separate cohort of middle-aged mice, exogenous introduction of *BDNF* to the hypothalamus via injection of an adeno-associated viral (AAV) vector was able to recapitulate much of the EE phenotype in mice with no exposure to EE. Of note, BDNF prevented the development of aging-associated metabolic decline and reduced anxiety- and depression-like behavior (McMurphy et al., 2018a). These observations further confirm the importance of the brain-body connection in systemic aging and implicate BDNF as an important player in healthy aging processes.

A considerable amount of research interrogates the relationship between neurological disease, environmental factors, and aging. EE has been shown to increase neurogenesis, dendritic complexity (Comery et al., 1996), dendritic spine number (Comery et al., 1996), synaptic plasticity (Leal-Galicia et al., 2008; Consorti et al., 2019), and cognitive flexibility (Gelfo, 2019), signifying the potential of the experimental model to promote healthy brain aging and slow the progression of neurodegenerative diseases. Previous work has described the relationship between EE and aging-related neurodegenerative disease in greater detail than can be discussed within the scope of this review (Nithianantharajah and Hannan, 2006; Laviola et al., 2008; Pang and Hannan, 2013; Mo et al., 2015; Shepherd et al., 2018). Perhaps unsurprisingly, EE has been shown to mitigate neurodegeneration observed in murine models of Alzheimer’s disease (Jankowsky et al., 2005; Lazarov et al., 2005; Cracchiolo et al., 2007), Parkinson’s disease (Faherty et al., 2005; Wassouf and Schulze-Hentrich, 2019), and Huntington’s disease (van Dellen et al., 2000; Hockly et al., 2002; Spires et al., 2004; Zajac et al., 2010). While the mechanisms of such diseases remain a topic of debate, future work with EE may reveal therapeutically relevant neuroprotective pathways that can be implicated in healthy aging.

Additional work by our lab has shown the importance of microglia in the aging-EE phenotype (Ali et al., 2019). Long-term EE (7.5 months) was shown to increase a microglial marker (*Iba1*) within the hippocampus, hypothalamus, and amygdala. Quantification of immunohistochemistry revealed that EE increased microglial hypertrophy and ramification, with no concurrent increase in microglial cell density. These alterations were observed alongside an EE-induced reduction in neuroinflammatory markers (*Ccl2, Il1b, Il6, Nfkbia, Socs3*, and *H2Ab1*) and improvement of systemic metabolism. Typically, aged microglial morphologies are described as de-ramified and are associated with altered inflammatory cytokine production (Sierra et al., 2007; Rawji et al., 2016). Our data suggests that EE can ameliorate many age-induced changes in microglial function, perhaps contributing to systemic health. Current work in our lab using functional depletion of microglia within older mice suggests that aged microglia contribute to age-related metabolic decline, perhaps through BDNF-dependent or BDNF-independant pathways.

### Caveats of Using EE to Study Lifespan

While our work and others’ suggest that complex environmental stimuli play an important role in promoting healthy aging, their relationship to lifespan is less conclusive. One experiment performed by our lab looked at the potential for EE to increase murine lifespan. Interestingly, a trending, but non-statistically significant increase in mean lifespan was observed when EE was initiated at 18 months of age (McMurphy et al., 2018b). Although EE may only have a mild impact on lifespan, it is encouraging that readily observable improvements to healthspan through EE are sustainable throughout age in our models. As lifespan studies are time and resource intensive, special care must be taken during experimental design process to anticipate the unexpected, and more work is needed to understand the EE-driven influence – or lack of influence – on murine lifespan. Careful integration of both healthspan and lifespan data will be important to discover mechanisms underlying healthy aging; as discussed previously, increased lifespan without concurrent improvements in healthspan may be problematic. For experiments interrogating the lifespan-environment interplay, large sample sizes and/or additional experimental challenges – like Western diet – may not only help ensure proper statistical power, but may also be more representative of human populations which may gain the most from similar interventions.

In sum, a great breadth of literature describes the ability of this dynamic environmental model to ameliorate or prevent age-related bodily dysfunction and disease. EE has been shown to increase healthspan and prompt healthy aging at least in part via hypothalamic induction of BDNF, highlighting the important connection between CNS and peripheral tissue function. While work on EE’s influence on lifespan is less conclusive, the observed improvements in systemic health underlie the necessity of understanding mechanisms inherent to the aging process. A better understanding of these processes may reveal therapeutically relevant targets to limit the negative aspects of aging and increase overall health, and thus further work in these areas is highly justified.

## The Search for EE Translation

Recent increases in global longevity underscore the need for improvements in healthspan. Without such improvements, aging individuals will spend a larger portion of their lives afflicted by acute and chronic disease, thus posing a large burden for healthcare providers. Therefore, it is important to understand the biological mechanisms by which healthspan can be increased. Previous sections in this review have commented on the nature of laboratory animal EE as a model for improving overall physical wellbeing, extending healthspan, and revealing biological mechanisms implicit in the brain-body connection. While EE has been well characterized by many groups, its translatability to humans has been a topic of debate (Burrows et al., 2011), perhaps due to the difficult task of connecting animal toys, shelters, bedding, etc. to the human condition. Complicating the matter, each animal in EE experiences a different combination of complex stimuli, shaping its phenotypic response (Kempermann, 2019). It is important to note that some scholars argue EE is more analogous to the normal human experience than the minimal physical, social, cognitive, motor, and somatosensory stimulation conferred by standard laboratory housing (Burrows et al., 2011). Indeed, some posit that EE might reverse a so-called “impoverishment” experienced within standard laboratory housing (Kempermann, 2019). With these positions in mind, we argue that EE might be a more apt model for understanding human aging than standard housing alone, providing a modeling system for inter-individual differences in exposure to complex stimuli that more closely mirrors the complexity of the human experience.

Detractors of the model suggest that EE introduces unnecessary cost, overhead, labor, and variability to experimental research (Hutchinson et al., 2005). Admittedly, variations between protocols exist, but some evidence suggests that between-laboratory effects contribute relatively little to total variance observed in EE experiments (Wolfer et al., 2004). It is our opinion that the main goal of EE is to reveal therapeutically-relevant, conserved biological mechanisms – indicated through brain-peripheral tissue axes – that can be implicated in human health and disease. Thus, specific technical differences between enrichment paradigms among laboratories or between murine models and humans which result in similar phenotypes are of minor consequence, and actually highlight the strength and robustness of the EE model system.

While the majority of biomedical research emphasizes discrete disease processes, a renewed focus on systemic processes like aging is warranted. In doing so, researchers will be able to consider biological mechanisms implicit in several comorbid disease states that accompany aging. Although EE allows for interrogation of such systemic changes in laboratory animals, the ultimate goal is to seek human therapeutics that modulate the same pathways revealed via EE, whether through lifestyle intervention efforts or targeted pharmacotherapies (Hannan, 2020). Here, we will discuss the search for lifestyle interventions that elicit similar biological pathways to those observed in laboratory EE. By integrating knowledge garnered from the EE model and human lifestyle interventions, we can begin to bridge the bench-to-bedside gap and thus begin the journey toward maximizing human healthspan.

### Physical Activity

Physical activity serves as one such example of a lifestyle intervention – it is known to restrict various age-related disease processes, including type II diabetes (T2D) (American Diabetes Association, 2004; Colberg et al., 2010; Aune et al., 2015), cardiovascular disease (CVD) (Wannamethee and Shaper, 2001; Mora et al., 2007), obesity (Wadden and Bray, 2018), and ischemic stroke (Kiely et al., 1994; Lee et al., 2003), among others. While physical activity becomes more difficult with age due to biological processes – including sarcopenia, arthritis, and osteoporosis – the benefits on systemic health cannot be ignored. Voluntary exercise is thought to modulate HPA axis signaling (Droste et al., 2003), immune function (Vieira et al., 2009; Pedersen et al., 2016), muscle maintenance (White et al., 2016), and adipose remodeling (Vieira et al., 2009; Stanford et al., 2015).

In preclinical studies across healthy, obese, and aging models, physical activity has been shown to increase *Bdnf* levels within central and peripheral tissues (Gómez-Pinilla et al., 2002; Molteni et al., 2004; Stranahan et al., 2009; Erickson et al., 2012). One group showed that voluntary exercise was protective against stress-induced decreases in BDNF (Adlard and Cotman, 2004). Voluntary exercise in murine longevity models shows clear healthspan benefits through central and peripheral neuroendocrine signaling, but data investigating the causal link between increases in lifespan after voluntary exercise are limited (Holloszy et al., 1985; Kujala, 2018).

It is important to note that a great deal of research has sought to define to what extent voluntary physical activity contributes to the EE phenotype in preclinical models. Some evidence indicates that voluntary physical activity and EE work in dissociable pathways (Olson et al., 2006). Additional work suggests that voluntary physical activity may act to “prime” physiological change induced within EE (Kronenberg et al., 2006; Fabel et al., 2009). Several reviews cover these topics in greater detail (Bekinschtein et al., 2011; Pang and Hannan, 2013; Rogers et al., 2019). We would like to especially note that the full EE phenotype often surpasses what is accomplished with exercise alone (Fabel et al., 2009; Cao et al., 2010, 2011; McMurphy et al., 2018b), suggesting the confluence of EE stimuli are vital for the observed improvements in health. In conclusion, physical activity is one lifestyle intervention that induces biological change inherent to healthy aging and may contribute to a compression of morbidity.

### Lost in Translation: Current Limitations of EE Crosstalk

In the laboratory setting, enrichment of an animal’s environment increases mental stimulation by providing access to exercise, additional peers for improved socialization, and novelty through regular rearrangement of environment. However, emergent properties of human intelligence and social structure compared to murine models means that more complex applications of these stimuli might not only be required to elicit beneficial psychological effect, but possibly necessary at a baseline level to achieve adequate health. For example, recognition of the importance of stimulation and inspiration by art as being vital, and not just optional, to the human experience has led several museums in the United States and Europe to provide free or reduced admission to those receiving social assistance. Additionally, many humans tend to actively seek out inherently complicated careers or hobbies, engage in spiritual or religious activities, or appreciate auditory and visual aesthetics difficult to translate into animal models. What a human may perceive as beauty, another animal may ignore or avoid. Furthermore, the effects of a particular stimulus can differ not only among individual people but also within a particular person as time goes on. For humans, it is difficult to tell where instinct ends and personality begins.

While many EE mechanisms such as BDNF, leptin, adiponectin, and the HPA axis stress response are known to be conserved between mice and humans, there may exist additional mechanisms unique to human-centric stimuli. If the mechanisms to these are wholly conserved, laboratory EE models will show even more promise to benefit humans. Alternatively, any differences will suggest additional mechanisms to target and induce in murine or other models, presenting ample future directions and assisting in effective human translation.

## Therapeutic Value of Enrichment and Lifestyle Interventions

Thus far, we have described how EE is uniquely positioned to interrogate the systemic processes that underlie aging, due in part to the ability of the model to confer global improvements in metabolism, immunity, and behavior that run counter to age-related systemic dysfunction. Additionally, we have discussed the so-called “translation problem” and have discussed the search for human lifestyle interventions that may elicit similar biological pathways to those observed in the EE paradigm. Below, we will discuss the potential therapeutic value of EE and lifestyle interventions to increase overall healthspan and how the model may inform future public health and medical considerations as researchers and clinicians connect the bench and bedside.

### Loneliness and Social Prescribing

As previously discussed, laboratory EE elicits various changes in the CNS that can manifest in improved behavioral health. While murine models cannot mirror complex human emotionality, we can consider the therapeutic value that EE analogs may provide in human mental health. For example, social partners are important for both laboratory animals and humans alike. Upon aging, humans experience a deterioration of social ties and some experience loneliness. While social isolation and loneliness have subtle differences – better discussed elsewhere (Hawkley and Cacioppo, 2007) – a plurality of research suggests that both may result in the manifestation of physical disease and mental illness – due to biological mechanisms induced by stress and coping mechanisms (Hawkley and Cacioppo, 2007). Concerns regarding loneliness in aged individuals have grown in recent years. In 2018, United Kingdom Prime Minister, Theresa May, appointed a “Minister for Loneliness” to address the growing public health concern of loneliness within the aging population. Over the coming years, a government initiative is set to connect lonely individuals with social activities, including cooking classes, art clubs, and walking clubs. Such efforts are described as “social prescribing” to stem the physical and mental toll that loneliness takes on aging individuals. Herein lies one public health initiative to imply therapeutic benefit for lonely aged individuals. While we cannot speculate on specific biological mechanisms here, such an initiative provides fodder for future research that interrogates whether such public health programs are beneficial and how systemic biological processes may be beneficially altered in engaged individuals.

### Cognitive Decline and Reserve

Aging leads to reduced cognitive function; however, the brain is resilient, having multiple coping mechanisms and reserve factors to combat the natural aging process. The cognitive reserve theory posits that the brain has an innate ability to cope with cerebral damage to minimize clinical manifestations of diseases like dementia and Alzheimer’s disease (Stern, 2002; Solé-Padullés et al., 2009); with a greater cognitive reserve buffer, more damage can be sustained before symptomatic presentation. In 2002, Stern characterized this idea and would later suggest that intellectual activities, like reading newspapers or books, could contribute to increased cognitive reserve capacity and thus reduced risk for manifestation of neurodegenerative disease (Stern, 2002; Scarmeas and Stern, 2003). Accordingly, environmental and lifestyle factors are considered key modulators of cognitive reserve (Scarmeas and Stern, 2003; Gelfo, 2019). Epidemiological work appears to confirm this notion (Stern and Barulli, 2019), identifying education as a key environmental factor that may contribute to increased cognitive reserve (Meng and D’arcy, 2012), as well as occupational status (Stern et al., 1994), smoking abstinence (Flicker et al., 2011), and regular physical activity (Rovio et al., 2005). An open area of exploration is how environment-driven increases in cognitive reserve might contribute to increased “mind-span,” or the length of an individual’s healthy cognitive function. In conclusion, the cognitive reserve theory runs alongside many of the discussions in this review and highlights another viewpoint regarding healthy aging and the importance of maintaining healthy environmental stimuli.

### Optimization of Environment in Medicine

Anecdotally, most people would agree that physical environment and psychological stress can affect recovery from disease or injury. However, it wasn’t until an article published in Science in 1984 by Ulrich (1984) that rigorous scientific study of the effects of design in the healthcare environment was truly considered. In 2008, an extensive literature review by Ulrich et al. (2008) deeply examined how exposure to factors such as natural and artificial light, noise, plants, art, air quality, color, simulated and actual views of nature, and support groups or other social opportunities could greatly affect the healing process. Exploration, categorization, and evaluation of these factors over the last 35 years has led to the development and iterative refinement of frameworks for “evidence based design” and best practices in which to create “optimal healing environments,” both inside and out of clinical encounters (Ulrich, 1991, 1997; Sakallaris et al., 2015; DuBose et al., 2018).

Early classifications of features required for healing predominantly focused on the perspective of a patient’s external environment including a sense of control of surroundings, access to social support, and access to positive distractions (Ulrich, 1991). Over time, others expanded and refined these classifications to evaluate the internal, interpersonal, and behavioral environments, in addition to external experiences of patients (Sakallaris et al., 2015). A recent review of the emotional, psychological, social, behavioral, and functional antecedents of healing has specifically indicated that creation of a homelike environment, with access to views and nature, appropriate light exposure, noise control, and a room layout that minimizes barriers not only improves patient safety and satisfaction, but also improves recovery, representing the emphasis that evidence based design places on environmental factors (DuBose et al., 2018). More specific explorations of these factors include studying patient exposure to natural and artificial light on sleep quality and subsequent recovery (Hadi et al., 2019), how the design of mental health facilities affects stress and behavior (Connellan et al., 2013; Ulrich et al., 2018), and how perceptions of loneliness diminish recovery from stroke (Anåker et al., 2019). Studies evaluating the effects of acoustics and sound on medical outcomes (Blomkvist et al., 2005; Joseph and Ulrich, 2007; Ulrich, 2008) have also concluded that sound can both help and hinder recovery. While ambient noise from poor building design, conversations heard from hallways or shared patient rooms, and various machines and medical devices negatively impact patients through impaired sleep and increased stress, relaxing music and music of a patient’s choice have been demonstrated to aid healing and decrease stress.

One underlying factor acknowledged in all of these studies and reviews is the significance of a patient’s perception of control, a factor considered important early on in this field (Ulrich, 1991). Even when both patients and healthcare providers ultimately have little to no control over outcomes, patients benefit from feeling like they can exert some influence over their situation. In a study exploring how perceptions of the treatment environment in a palliative care setting affected patient perceptions of positive emotion during treatment (Timmermann et al., 2014), patients reported that personalized decorations, maintaining a familiar daily rhythm with familiar tasks, and creation of a sense of “coziness” or “homeliness” was important for their sense of well-being and experience of positive emotions. It is clear that these factors do not only impact terminally ill patients and that the contribution of environmental factors on psychological and physical health extend to people in all states of health.

Another well explored aspect of improving health and the healing process is exposure to nature. Instead of incidentally viewing nature through a window, patients and their families are now receiving in-depth real and simulated exposure in and out of the healthcare setting surpassing just a therapeutic adjunct. Recent work in this area has included studying psychological benefits from exposure to nature through indoor and outdoor spaces in general, Lacanna et al. (2019), Sachs (2019), as well as during childbirth (Aburas et al., 2017), before colonoscopy (Sjölander et al., 2019), for those visiting someone in an intensive care unit (Ulrich et al., 2019), and exposure to sights, sounds, and scents from a traditional Japanese garden for those with cognitive impairment (Goto et al., 2017). These studies and reviews emphasize that exposure to nature decreases stress, increases happiness and satisfaction with treatment, improves sleep, and leads to measurable improvements in recovery; additionally, these exposures provide socialization and an improved perception of control of one’s environment. It is important to consider the complexity of healthcare environments in such studies; care must be taken to evaluate whether these nature-related stress reductions are correlative or causative, controlling for relevant variables. The development and use of biomarkers will push this field forward, alongside clinical outcome metrics.

It is important to iterate that the therapeutic benefits of factors useful in creating optimal healing environments are not limited to healthcare settings or even in unhealthy individuals. More recent work has explored the use of nature-based stimuli as a therapeutic driver for well-being in healthy aged individuals (Gagliardi and Piccinini, 2019; Gagliardi et al., 2019; Santini et al., 2019). Horticultural therapy within community or home gardens and the inclusion of green spaces within neighborhoods, hospitals, and nursing homes shows incredible promise to benefit people across a wide spectrum of health. Experiencing complex stimuli such as nature shows immense benefits to one’s well-being, in sickness and in health, and even when simulated through pictures or video or through temporary exposure.

## Discussion and Future Considerations

In this review, we have presented EE (Figure 1) as an apt model for studying healthspan and have speculated on how we might begin to translate findings to the clinic in search of extended healthspan. The EE model is exceptionally well poised to probe questions within the aging research field, as EE’s complex stimuli mirror those that are thought to elicit healthy aging processes in humans and thus elicit maximization of healthspan. While the translatability of EE is a topic of debate, we argue that the model’s importance lies within its ability to reveal therapeutically-relevant mechanisms that underlie the brain-body connection, and thus global biological processes.

Although the exact stimuli in laboratory EE differ among research groups or between murine models and humans, EE models provide a starting point for human enrichment and subsequent improvements to health and well-being. In fact, the ability for similar sustained and robust results to occur despite differences further highlights the strengths of the model and the importance of the underlying physiology. The fundamental mechanisms such as hypothalamic BDNF, leptin, adiponectin, and the HPA stress response, are conserved. Despite the ability to study and manipulate laboratory models of EE, researchers are limited in measuring or modulating these systems in humans; for example, we and others cannot biopsy brain tissue in living humans to measure gene or protein expression or observe certain changes to organ structure and function. Further research in aging and healthspan through EE in laboratory models would strongly benefit from additional exploration and identification of potential biomarkers or other measurable factors specific to enrichment or healthspan so that we may better understand and evaluate effects of potential enrichment paradigms on humans. Many of the human studies and reviews referenced in previous sections acknowledged the potential for using blood pressure, pulse, and serum or salivary cortisol to more objectively measure the benefits of a proposed intervention. We similarly believe that human studies would immensely benefit from these and other objective measurements of physiological change to measure the actual effects of enrichment.

We also acknowledge the dual diagnostic and prognostic potential of using biomarkers. Measurement and evaluation of biomarkers might not only be useful in measuring the efficacy of a lifestyle intervention, but may also assist in determining which people would most or least benefit from a particular intervention, creating an opportunity for more personalized and cost-effective care. Some have proposed that epigenetic clocks could act as an advanced biomarker for assessing lifestyle intervention efficacy, however DNA methylation is not thought to be a particularly dynamic marker of short-term lifestyle changes (Horvath and Raj, 2018). Work in this field is ongoing; early evidence supports an association between lifestyle factors – including diet, education, physical activity, and low BMI – and epigenetic age (Quach et al., 2017).

Additional research toward identification of aging biomarkers is ongoing and will require next-generation –omics strategies for identification of new targets. One meta-analysis suggests several biomarkers – total adiposity, visceral adiposity, CRP, IL-6, insulin, IGF-1, HOMA, adiponectin, leptin, and IGF binding proteins – to assess efficacy of dietary interventions in aged individuals (Lettieri-Barbato et al., 2016). Others have characterized serum biomarkers of DNA damage – CRAMP, EF-1α, stathmin, *n*-acetyl-glucosaminidase and chitinase – and have shown lifestyle interventions to be effective in altering these markers (Song et al., 2010). While several of these biomarkers would be considered by some to be the “usual suspects,” the discoveries of so-called “geroprotective footprints” (Lettieri-Barbato et al., 2016) or “healthy aging phenotypes” (Lara et al., 2013) will be essential to bridge bench and bedside work.

While we know that the combination of complex physical, social, cognitive, motor, and somatosensory stimuli within EE can elicit global changes in aged metabolism, immunity, and behavior, there is still a great deal of opportunity for more research. Work in young mice indicates that EE confers improvements in innate and adaptive immunity; we are currently working to determine whether aged mice exhibit similar results in the face of a cancer challenge. Similar experiments interrogating the role of the HPA axis in the EE-aging phenotype are needed. Furthermore, the field needs further work regarding the effect – or lack of effect – that EE may have on murine lifespan; such research will require careful design and large sample sizes.

While BDNF has been implicated as one important molecular mediator of healthy aging, additional work is needed to elucidate central, systemic, and peripheral actors that might yield a compression of morbidity and thus extension of healthspan. Though exogenous introduction of BDNF in the human brain is an unlikely treatment for aging itself, animal experiments helped to elucidate BDNF as an upstream mediator of healthy aging processes. Such observations also emphasize the importance of the brain-body connection in systemic aging. Our hope is that these preclinical experiments provide the framework for understanding how human lifestyle interventions and environmental stimuli might initiate similar biological change toward healthspan extension and compression of morbidity. With these insights, researchers can develop novel environmimetics and epimimetics for pharmacological use – an exceptionally important goal because lifestyle interventions are difficult to implement in the long-term and are rarely a panacea (Kelly and Hannan, 2019; Hannan, 2020).

The aging process is highly heterogeneous and this is reflected through the vast differences in the physical and mental state of aged individuals. Of note, many aging processes occur in a sexually-dimorphic manner (Berchtold et al., 2008). Historical data shows that women outlive their male counterparts, even when controlling for cultures and causes of death (Austad, 2006). Despite this, women have higher overall rates of physical illness, disability days, chronic illnesses, and hospital stays than do men (Wingard, 1984). Animal modeling of aging-related sexual dimorphisms provides its own challenges. Notably, some animal models are incongruent with the human findings, with males more likely to live longer than females (Turturro et al., 1999). Historically, males have been the sex of choice for animal experiments, leading to biases in the literature. Unfortunately, a paucity of data exists in animal models to explain these the biology of aging sexual dimorphisms within EE. This is an ongoing field of study; naturally, we wonder if aging-EE phenotypes will be affected by sex. Our recent EE-aging work was completed in females (McMurphy et al., 2018b; Ali et al., 2019) for an experimental design reason; we surmised that they would be less likely to fight and compromise a long-term aging study. Further work investigating sexual dimorphisms in the aging-EE space is warranted, although special care must be taken to choose the appropriate animal model and experimental design.

While the majority of this review has covered the negative aspects of aging, some positivity can be found within the human aging process, which Rowe and Kahn termed “successful” aging. With age and retirement, some humans have more free time; while this time is fleeting, a greater portion is available for enjoyment of hobbies, traveling, and family or other social support systems. Aged humans also tend to better understand themselves in terms of strengths, weaknesses, and preferences. Rowe and Kahn recognized these and similar factors as contributing to a stronger autonomy and sense of control of one’s life as people age (Rowe and Kahn, 1987). However, the loss of support and control in one’s life from physical or mental ailments due to disease or normal aging, preclude maximization of this extra time and lead to further deterioration. This furthers the importance of determining biological mechanisms inherent to healthspan so that we might maximize precious time and happiness in our waning years.

Topics in this review suggest the essential role public health measures will play in implementation of healthy aging interventions. The aforementioned cognitive reserve theory indicates that some groups may be at higher risk for the development of neurodegenerative disease than others, due in part to reduced exposure to brain-stimulating activities. As we continue to explore both the breadth of application and the depth of known mechanisms involved in laboratory EE models, we will improve our ability to effectively apply their principles to humans. Current and future results from these efforts, in combination with public health initiatives and epidemiological evaluation, have great potential to improve human healthspan. Identification and engagement of at-risk populations will be essential in causing a noticeable shift in healthspan on a population level.

While these effects may take generations to become apparent, changes in the clinic are already occurring. There has been an increased focus on the “social determinants of health” over the last few decades (Schroeder, 2007) and a creation of “health systems science” to understand how to best deliver healthcare (Gonzalo et al., 2017a, b). To help accomplish this, allopathic medical schools in the United States have recently emphasized collecting more thorough social histories to aid in evaluation and treatment of a patient’s overall well-being and health. While medical fact-finding has long included inquiry of recreational hazards such as drug use or high-risk sexual activity, students are now taught to gather and consider detailed information on a patient’s diet, exercise, living situation, education, employment, recreation, involvement in their community, spirituality, and access to a sufficient support network. These data do more than just predict patient non-adherence; more importantly they help identify unknown factors impairing treatment. Knowledge of these environmental factors enable clinicians to discuss and engage in non-pharmacological treatments which may improve the effect of a treatment or possibly even reduce or eliminate its need. These didactic shifts indicate the acknowledgment of the influence of environment in clinical outcomes and the importance of a person’s psychological health toward their holistic health and well-being.

In summary, the goal to extend healthspan is a lofty one; researchers require a multitude of techniques to investigate which environmental interventions may have the greatest clinical impact. EE is one of the best-served tools due to its ability to ameliorate or prevent many age-associated declines in physiological function. In considering the brain-body connection, researchers can best understand the systemic processes that underlie aging and thus develop and evaluate effective therapeutic options to extend and improve healthspan.

## Author Contributions

NQ and QH wrote the review. LC revised the manuscript and provided the funding.

## Conflict of Interest

The authors declare that the research was conducted in the absence of any commercial or financial relationships that could be construed as a potential conflict of interest.
